# Supplementary material for: Ring Finger 149-Related Is an FGF/MAPK-Independent Regulator of Pharyngeal Muscle Fate Specification
Source: Int J Mol Sci. 2023 May 16;24(10):8865. doi: 10.3390/ijms24108865 (PMC10219245; doi:10.3390/ijms24108865)

Supplemental Material

Supplementary Table 1: Supp table 1.xlsx
Column legend:
asmFC18: Fold changes of genes that were significantly assigned to atrial siphon muscle cells at 18 hpf in scRNA-seq
fhpFC18: Fold changes of genes that were significantly assigned to first heart precursor cells at 18 hpf in scRNA-seq
shpFC18: Fold changes of genes that were significantly assigned to first second precursor cells at 18 hpf in scRNA-seq
asmFC20: Fold changes of genes that were significantly assigned to atrial siphon muscle cells at 20 hpf in scRNA-seq
fhpFC20: Fold changes of genes that were significantly assigned to first heart precursor cells at 20 hpf in scRNA-seq
shpFC20: Fold changes of genes that were significantly assigned to first second precursor cells at 20 hpf in scRNA-seq

Supplementary Table 2: Supp Table 2

**Hours post fertilization (hpf)**	**FABA Stage** (Hotta et al., 2007; Hotta et al., 2020)	**Developmental Stage**
12	Stage 23	late tailbud I
15	Stage 24/25	late tailbud II-III
18	Stage 26/27	hatching larva
20	Stage 28	early swimming larva
26	Stage 30	early swimming larva

Supplementary Table 3: Supp Table 3
Comprehensive list of *Ciona* proteins that have recognized PA-domains.
ENSCINP00000031058 maps to 2 KH IDs.

**ENSEMBLID**	**GeneID**	**TranscriptID**	**KHID**	**KYID**	**Gene Name**	**Best 3 Human Orthologues**
ENSCINP00000018401	ENSCING00000009069	ENSCINT00000018401	KH2013:KH.S215.3	KY2019:KY.Chr13.453	EDEM2/3	EDEM1; EDEM2; EDEM3
ENSCINP00000031058	ENSCING00000021112	ENSCINT00000031264	KH2013:KH.C14.482	KY2019:KY.Chr14.219	KH2013:KH.C14.482	
			KH2013:KH.C8.229	KY2019:KY.Chr8.448	NAALAD2	FOLH1; NAALAD2; NAALADL1

ENSCINP00000010505	ENSCING00000005093	ENSCINT00000010505	KH2013:KH.C14.568	KY2019:KY.Chr14.218	GTF2E2	T2EB
ENSCINP00000034768	ENSCING00000018380	ENSCINT00000036660	KH2013:KH.C11.425	KY2019:KY.Chr11.1091	VPS70	FOLH1; NAALAD2; NAALADL1
ENSCINP00000014713	ENSCING00000007174	ENSCINT00000014713	KH2013:KH.C7.451	KY2019:KY.Chr7.828	KH2013:KH.C7.451_FOLH1	FOLH1; NAALAD2; NAALADL1
ENSCINP00000011038	ENSCING00000005372	ENSCINT00000011038	KH2013:KH.S346.8	KY2019:KY.Chr2.2186	C35C5.2	FOLH1; NAALAD2; NAALADL1
ENSCINP00000023283	ENSCING00000012489	ENSCINT00000023529	KH2013:KH.C1.401	KY2019:KY.Chr1.2172	RNF13	RN167; RNF13; ZNRF4
ENSCINP00000021924	ENSCING00000011483	ENSCINT00000022170	KH2013:KH.C2.730	KY2019:KY.Chr2.1035	KH2013:KH.C2.730_RNF149	RNF130; RNF149; RNF150
ENSCINP00000030411	ENSCING00000011483	ENSCINT00000035820	KH2013:KH.C11.361	KY2019:KY.Chr11.1266	RNF215	GOLI; RN150; RN215
ENSCINP00000022045	ENSCING00000011573	ENSCINT00000022291	KH2013:KH.C2.994	KY2019:KY.Chr2.519	KH2013:KH.C2.994_RNF149	RNF130; RNF148; RNF149
ENSCINP00000030577	ENSCING00000023464	ENSCINT00000030308	KH2013:KH.C4.37	KY2019:KY.Chr4.430	PRADC1	PADC1

Supplementary Table 4: Genes tested and primers used to construct sgRNAs to validate post-transcriptional regulators detected by scRNA-seq.

**Transcript (KH) ID**	**Gene Name**	**Oligo name**	**sequence 5'-3'**
KH2013:KH.C2.994	Rnf149-r	sgRNF_2_fw	ggatggtggacctgacacatgtttaagagctatgctggaaacag
KH2013:KH.C2.994	Rnf149-r	sgRNF_2_rev	atgtgtcaggtccaccatccatctataccatcggatgccttc
KH2013:KH.C2.994	Rnf149-r	sgRNF_4_fw	ggagttacactgttaagaaggtttaagagctatgctggaaacag
KH2013:KH.C2.994	Rnf149-r	sgRNF_4_rev	cttcttaacagtgtaactccatctataccatcggatgccttc
KH2013:KH.C10.250	RBFOX1/2/3	sgRBFOX_1_F	ggagttctgcatgtaggtgggtttaagagctatgctggaaacag
KH2013:KH.C10.250	RBFOX1/2/3	sgRBFOX_1_R	ccacctacatgcagaactccatctataccatcggatgccttc
KH2013:KH.C10.250	RBFOX1/2/3	sgRBFOX_4_F	ggcttgaacgaattcagctggtttaagagctatgctggaaacag
KH2013:KH.C10.250	RBFOX1/2/3	sgRBFOX_4_R	cagctgaattcgttcaagccatctataccatcggatgccttc

KH2013:KH.C7.205	ASB2	sgASB_1_F	gaaagtttggaggaatcgctgtttaagagctatgctggaaacag
KH2013:KH.C7.205	ASB2	sgASB_1_R	agcgattcctccaaactttcatctataccatcggatgccttc
KH2013:KH.C7.205	ASB2	sgASB_2_F	gggtggcataagtgatgagtgtttaagagctatgctggaaacag
KH2013:KH.C7.205	ASB2	sgASB_2_R	actcatcacttatgccacccatctataccatcggatgccttc
KH2013:KH.C7.205	ASB2	sgASB_6_F	gacctacgtaacatcgattggtttaagagctatgctggaaacag
KH2013:KH.C7.205	ASB2	sgASB_6_R	caatcgatgttacgtaggtcatctataccatcggatgccttc
KH2013:KH.L9.27	RBM24/38	sgRBM_1_F	gtacaccaagatattcgtgggtttaagagctatgctggaaacag
KH2013:KH.L9.27	RBM24/38	sgRBM_1_R	ccacgaatatcttggtgtacatctataccatcggatgccttc
KH2013:KH.L9.27	RBM24/38	sgRBM_6_F	gatccgaacccggtgatcgagtttaagagctatgctggaaacag
KH2013:KH.L9.27	RBM24/38	sgRBM_6_R	tcgatcaccgggttcggatcatctataccatcggatgccttc
KH2013:KH.S115.4	QKI	sgQKI_5_F	gcctgctggtggtagttatggtttaagagctatgctggaaacag
KH2013:KH.S115.4	QKI	sgQKI_5_R	cataactaccaccagcaggcatctataccatcggatgccttc
KH2013:KH.S115.4	QKI	sgQKI_6_F	gagcgccgttggtggtaggcgtttaagagctatgctggaaacag
KH2013:KH.S115.4	QKI	sgQKI_6_R	gcctaccaccaacggcgctcatctataccatcggatgccttc
KH2013:KH.C11.417	NOVA1/2	Nova1.2_F	gacaggcctacacggcacagtttaagagctatgctggaaacag
KH2013:KH.C11.417	NOVA1/2	Nova1.2_R	tgtgccgtgtaggcctgtcatctataccatcggatgccttc
KH2013:KH.C11.417	NOVA1/2	Nova2.1_F	gaagaatgagctggcccgggtttaagagctatgctggaaacag
KH2013:KH.C11.417	NOVA1/2	Nova2.1_R	ccgggccagctcattcttcatctataccatcggatgccttc
KH2013:KH.C11.417	NOVA1/2	Nova2.3_F	gtaccggggtacgctgcgggtttaagagctatgctggaaacag
KH2013:KH.C11.417	NOVA1/2	Nova2.3_R	ccgcagcgtaccccggtacatctataccatcggatgccttc
KH2013:KH.C2.730	RNF150	sgRNF150_2_F	gtgttgtcacatggaattatgtttaagagctatgctggaaacag
KH2013:KH.C2.730	RNF150	sgRNF150_2_R	ataattccatgtgacaacacatctataccatcggatgccttc
KH2013:KH.C2.730	RNF150	sgRNF150_3_F	gggcttgctttggtttcacggtttaagagctatgctggaaacag
KH2013:KH.C2.730	RNF150	sgRNF150_3_R	cgtgaaaccaaagcaagcccatctataccatcggatgccttc
KH2013:KH.C2.730	RNF150	sgRNF150_5_F	gtagagacaatgagccatgcgtttaagagctatgctggaaacag
KH2013:KH.C2.730	RNF150	sgRNF150_5_R	gcatggctcattgtctctacatctataccatcggatgccttc

Supplementary Table 5: *Tyr*^{CRISPR} vs. *Rnf149-r*^{CRISPR} RNA-seq gene list with log fold-change (logFC), p-value (PValue) and false discovery rate (FDR) values.

toptags_rnfko_KH_UniqueName.xlsx

Column legend:

logFC: Log fold change, logCPM: Log counts per million, FDR: False Discovery Rate

Supplemental figure legends

Figure S1: FISH validation of the expression for candidate genes.
First row is predicted heart genes, second and third row predicted ASM genes. mRNAs are visualized by whole mount fluorescent in situ hybridization (green). Nuclei of cells are marked by Mesp>nls::LacZ and revealed by anti beta-galactosidase antibody (red). Mesp>hCD4::mCherry accumulates in the cell membrane and is revealed by anti mCherry antibody (blue). White line indicates the midline.

Figure S2: FISH validation and violin plots of the expression for *Rnf149* at different developmental time points.
a) mRNAs are visualized by whole mount fluorescent in situ hybridization (green). Cells are marked by *Mesp>mCherry*. b) Violin plots represent the distribution of the expression of indicated genes in defined cell clusters (Wang et al., 2019). Each cell is represented by a black dot. On the x-axis, the numbers indicate hours post fertilization (hpf). Scale bar, 50 µm.

Figure S3: Both sgRNAs used to generate *Rnf149-r*^{CRISPR} contribute to the phenotype.
a) Schematic of the *Rnf149-r* gene and the positions of the two sgRNAs used. b) Histogram with phenotype proportions. “n=” represents the total numbers of individual halves scored per condition. Error bars represent 95% Wilson method of confidence interval for proportions.

Figure S4: *Rnf149-r*^{CRISPR} does not alter the TVC marker *Hand-r* expression at 12 hpf.
Left panel shows the in situ hybridization against a *Hand-r* probe in green. *Tyr*^{CRISPR} used as control. Cardiopharyngeal lineage marked by mCherry driven by *Mesp* and revealed by mCherry antibody in red. H2B::mCherry accumulates in the nuclei. Scale bar, 50 µm. Right panel shows the corresponding histogram with phenotype proportions. “n=” represents the total numbers of individual halves scored per condition.

Figure S5: There are 11 PA domain-containing genes in the Ciona genome.
PA domains shown in light blue, RING domains shown in green, Zn-independent exopeptidase domains shown in yellow, transferrin receptor-like dimerization domains shown in red and seven-hairpin glycosidase domain shown in purple.

Figure S6: *Rnf150*^{CRISPR} did not result in significant phenotypic deformities at 26 hpf.
Histogram with phenotype proportions. “n=” represents the total numbers of individual halves scored per condition.

Figure S7: The histogram for the TVC-specific *FoxF* enhancer labeling, FoxF>LacZ , with phenotype proportions. “n=” represents the total numbers of individual halves scored per condition. Error bars represent 95% Wilson method of confidence interval for proportions.

Supplemental fig. 1

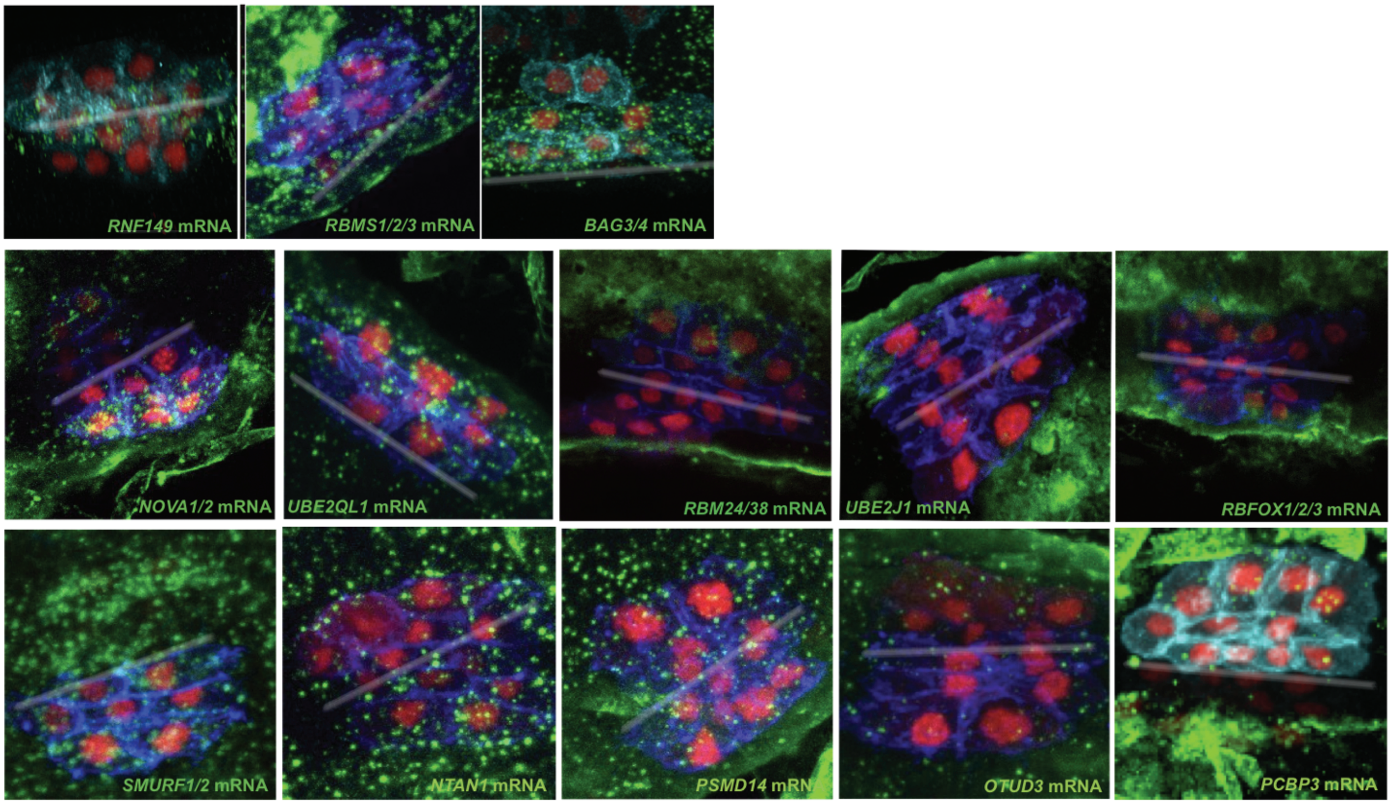

Supplemental fig. 2

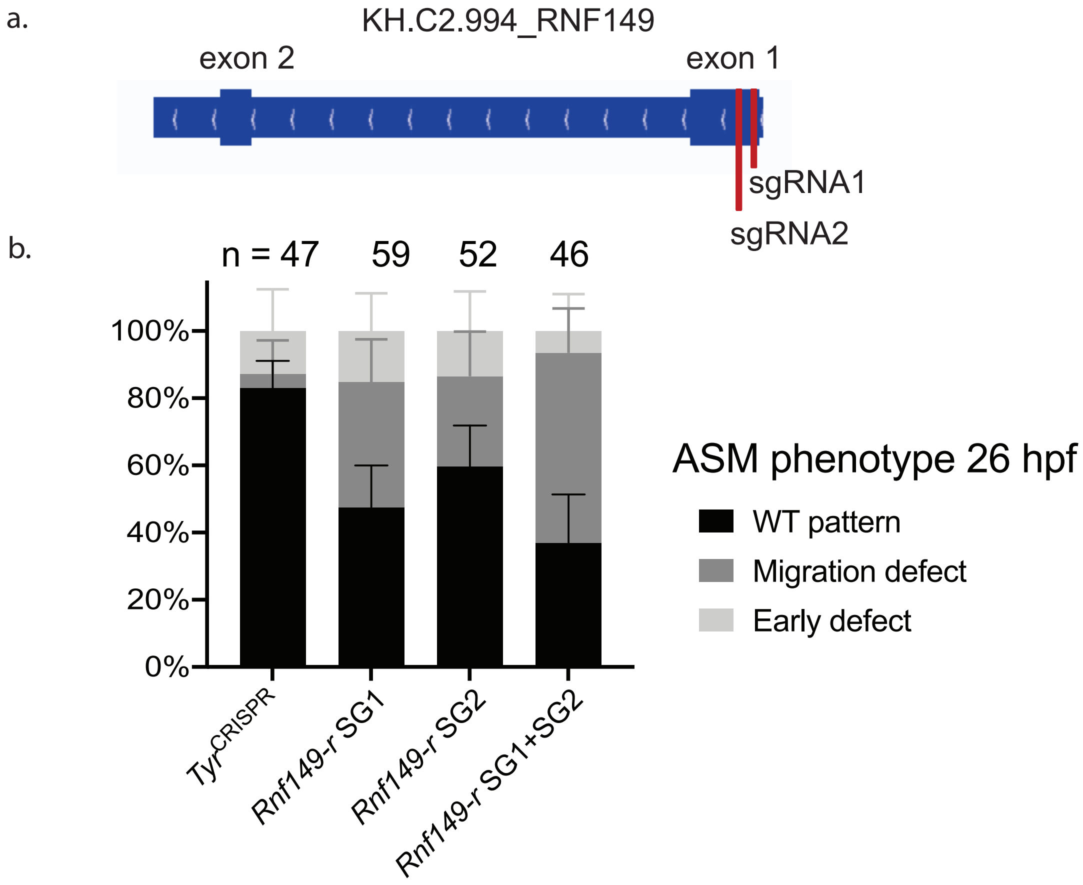

Supplemental fig. 3

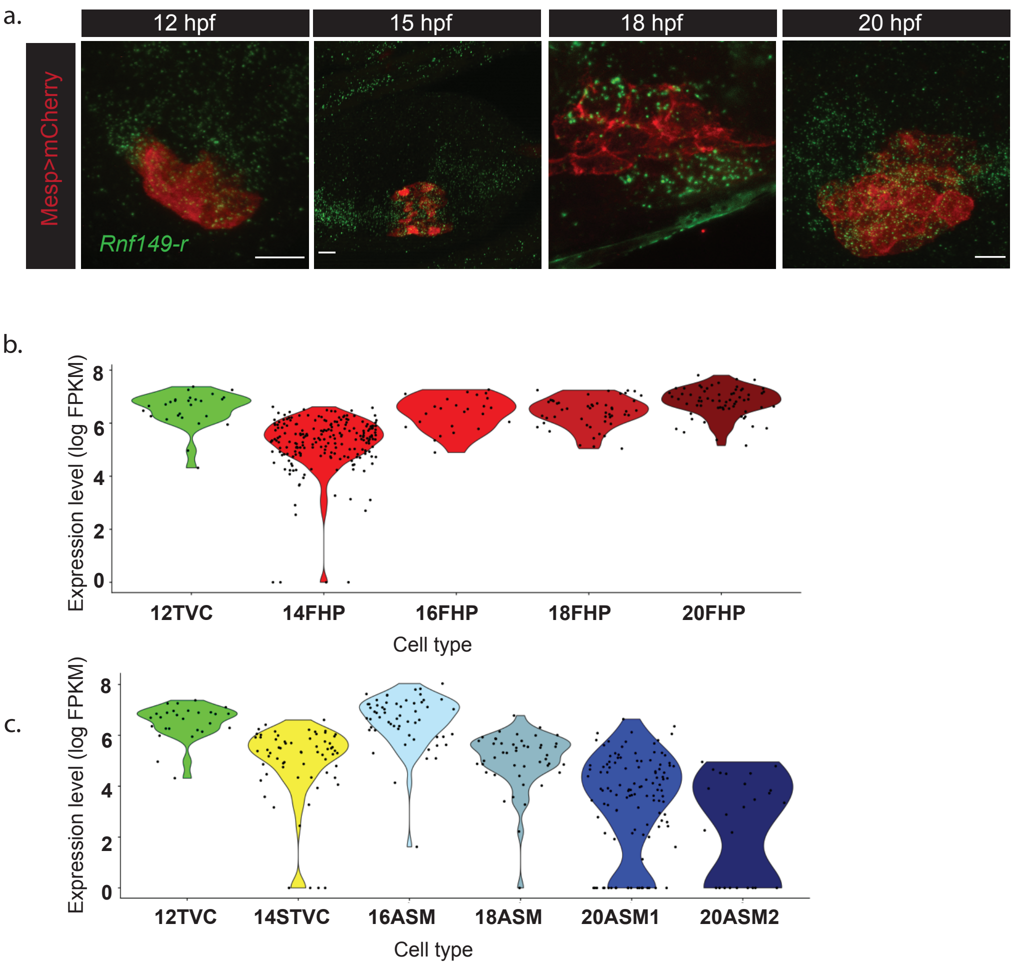

Supplemental fig. 4

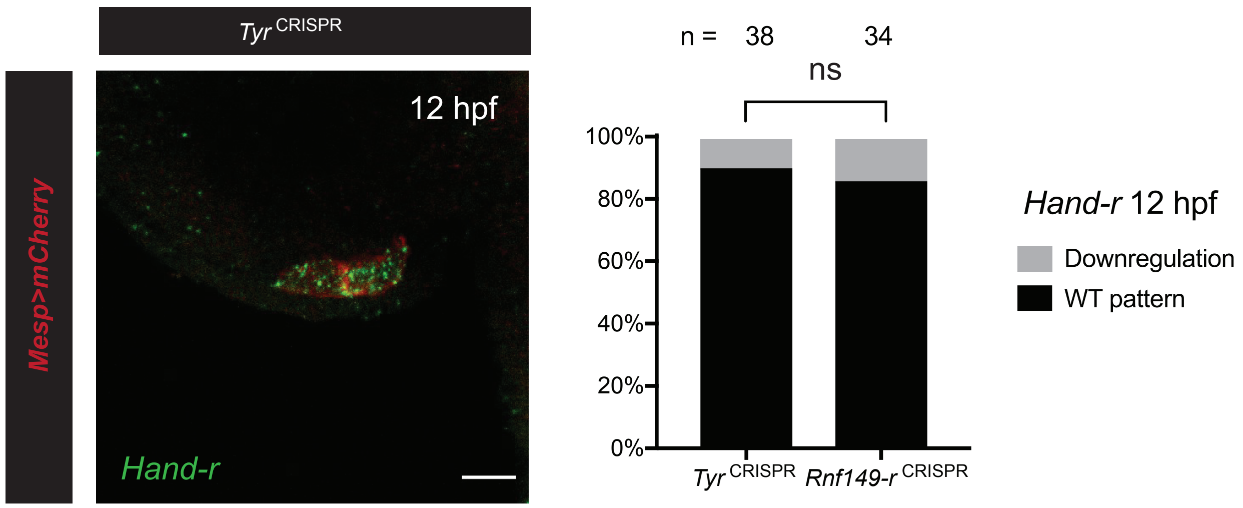

Supplemental fig. 5

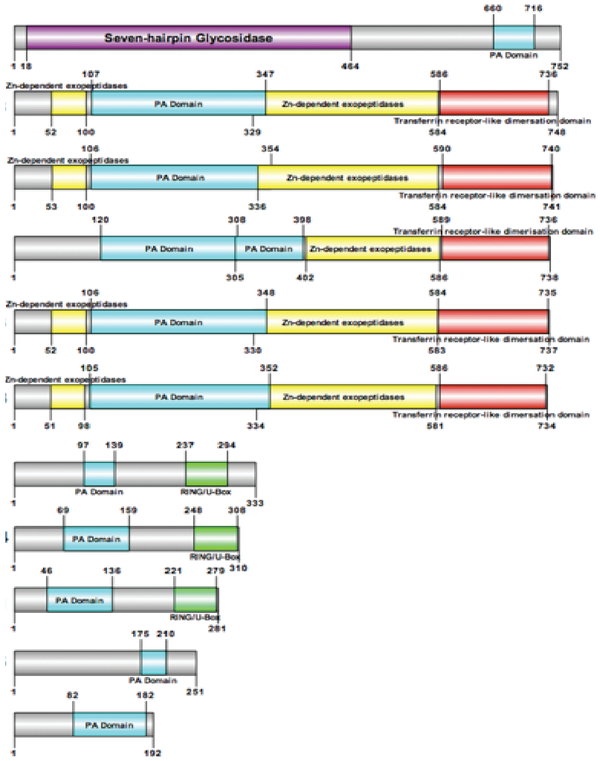

Supplemental fig. 6

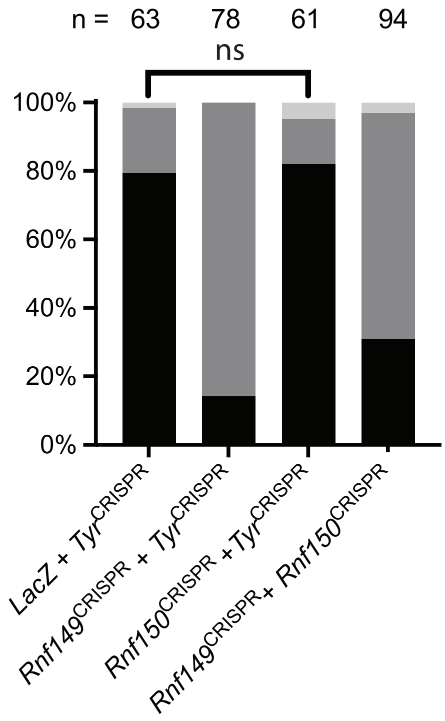

Supplemental fig. 7

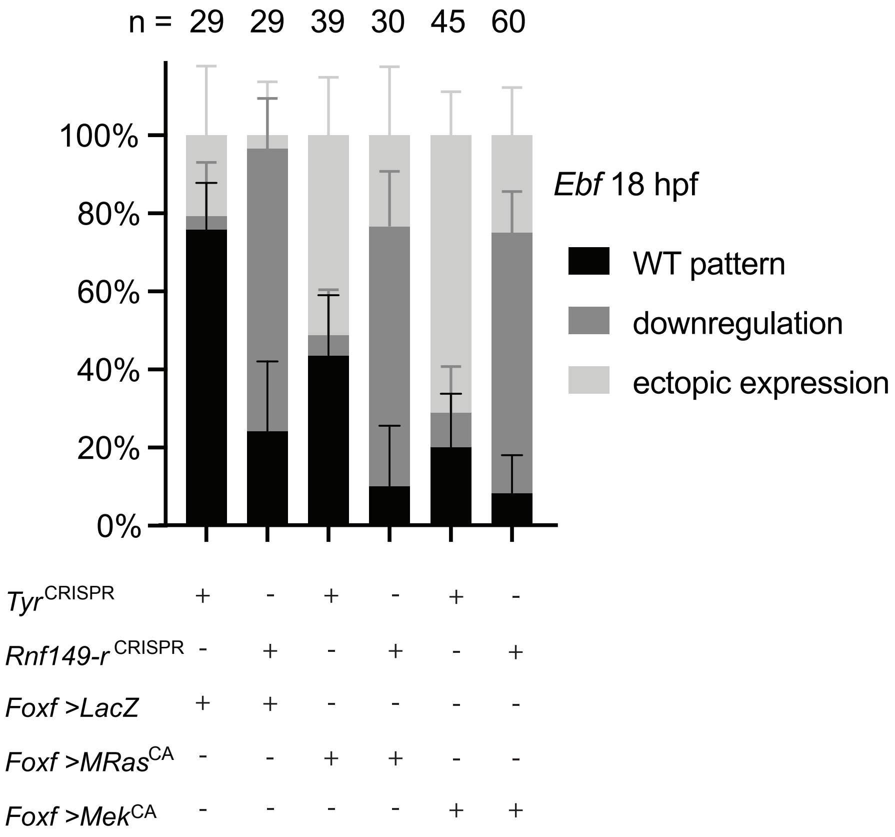

Supplement: Supplementary file 1 [file ijms-24-08865-s001.zip › ijms-2310188-supplementary.pdf]
